# Serine protease PRSS56, a novel cancer-testis antigen activated by DNA hypomethylation, promotes colorectal and gastric cancer progression via PI3K/AKT axis

**DOI:** 10.1186/s13578-023-01060-0

**Published:** 2023-07-03

**Authors:** Dandan Li, Lingyun Xia, Pan Huang, Zidi Wang, Qiwei Guo, Congcong Huang, Weidong Leng, Shanshan Qin

**Affiliations:** 1 Department of Stomatology, Taihe Hospital and Hubei Key Laboratory of Embryonic Stem Cell Research, School of Basic Medical Sciences, Hubei University of Medicine, Shiyan, Hubei 442000 P.R. China; 2 Laboratory of Tumor biology, Academy of Bio-Medicine Research, Hubei University of Medicine, Shiyan, Hubei 442000 P.R. China

**Keywords:** Cancer/testis gene, DNA methylation, Serine protease, Tumor antigen

## Abstract

**Background:**

Cancer/testis (CT) antigens/genes are usually overexpressed in cancers and exhibit high immunogenicity, making them promising targets for immunotherapy and cancer vaccines. The role of serine protease PRSS56 in cancers remains unknown to date.

**Methods:**

RNA sequencing studies were performed to screen CT genes in gastric cancer (GC) and colorectal cancer (CRC) cells exposed to DNA methyltransferase inhibitor 5-aza-2’-deoxycytidine (5-AZA-CdR). Bioinformatics analysis was conducted to analyze the correlation between PRSS56 expression and DNA methylation. Functional experiments were performed to explore the biological function of PRSS56 in GC and CRC.

**Results:**

In this study, we identified the testis-specific serine proteases PRSS56 as a novel CT antigen. PRSS56 was frequently overexpressed in various cancers, especially in gastrointestinal cancer. PRSS56 expression was negatively associated with promoter DNA methylation level, and positively associated with gene body methylation level. PRSS56 expression was significantly activated in colorectal and gastric cancer cells exposed to DNA methyltransferase inhibitors. Importantly, our finding highlights that the decreased methylation level of the CpG site cg10242318 in the PRSS56 promoter region resulted in its overexpression in GC and CRC. Additionally, functional assays verified that PRSS56 overexpression activated PI3K-AKT signaling in GC and CRC.

**Conclusion:**

Serine protease PRSS56 is a novel CT antigen that is reactivated in cancers by promoter DNA hypomethylation. PRSS56 functions oncogenic roles in GC and CRC by activating of PI3K/AKT axis. Our results presented here represent the first data on the function of the serine protease PRSS56 in cancers.

## Background

Cancer-testis (CT) genes or antigens are a class of genes that represent similarity between gametogenesis and tumorigenesis, play essential roles in tumor progression [1]. CT genes are selectively expressed in testis and rarely expressed in other tissues, but exhibit significant reactivation of the expression in cancers other than testis tumor [2]. The reactivation of CT antigens expression and high immunogenicity make them promising targets for immunotherapy in cancers [3]. Increasing studies have confirmed that the abnormal activation of CT genes plays critical roles in driving tumorigenesis and cancer metastasis [4–6]. Therefore, it’s necessary to identify novel oncogenic CT antigens in cancers, which may be of great significance for improving patient outcomes.

Serine protease is a kind of important proteolytic enzyme with serine as the active center, which plays an important and extensive physiological role in biological organisms, including digestion, blood coagulation fertilization, fibrinolysis, cell apoptosis and differentiation, angiogenesis [7]. According to different protein structures, serine proteases can be further divided into trypsin-like serine proteases (PRSSs) and type II transmembrane serine proteases (TTSPs) [8]. Emerging evidence has shown that serine proteases also play critical roles in tumorigenesis and metastasis [9]. Serine protease PRSS3 has been found to play oncogenic roles in pancreatic cancer, stomach cancer and prostate cancer [10–12]. Serine protease PRSS8 has been reported to play tumor-suppressive roles in colorectal cancer, bladder cancer and liver cancer [13–16].

As a trypsin-like serine protease, PRSS56 was found to play a role in development of eye and neurogenesis [17–19]. However, the biological function of serine protease PRSS56 in cancers remains unknown to date. In this study, we identified PRSS56 as a novel CT antigen that is frequently overexpressed in cancers, especially in GIC. Our finding highlighted that the upregulation of PRSS56 in GC and CRC was due to the decreased methylation level of promoter DNA. More importantly, our results suggested that PRSS56 overexpression promoted GC and CRC progression via the PI3K/AKT axis. Notably, our results presented here represent the first data on the function of the PRSS56 in human cancer.

## Results

### Landscape of expression pattern of 42 serine proteases in human tissues

**Fig. 1:**
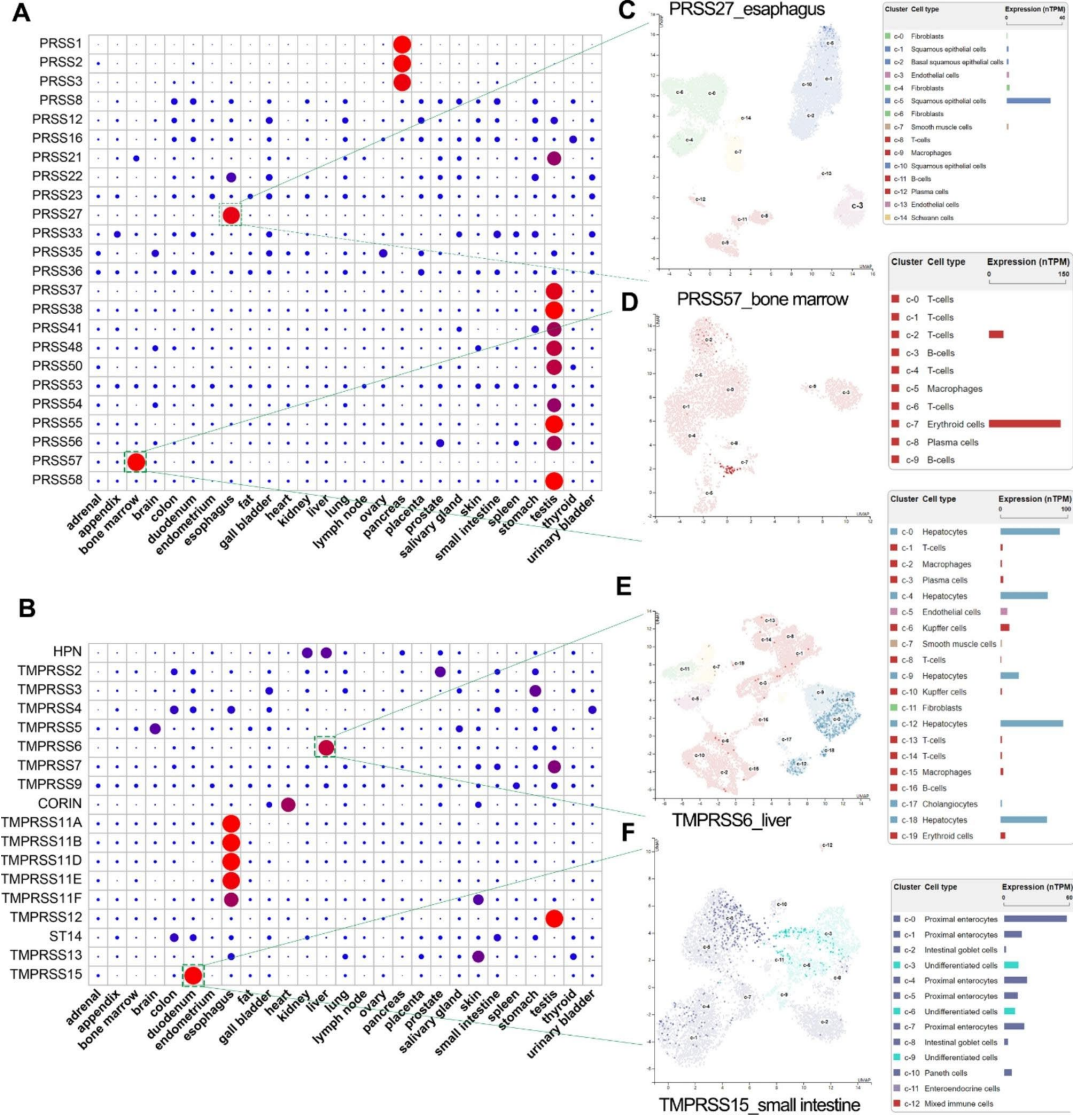
The expression pattern of 42 serine protease in human tissues. (A and B) The tissue-specific expression analysis of 24 PRSS genes and 18 TMPRSS genes. The expression data (RPKM value) of 42 serine proteases in various human tissues was available from the HPA project using the NCBI website. The dot plot is generated using relative expression value of each serine protease. In short, the relative expression value of each gene was obtained by making the sum of the RPKM value of each gene in all tissues be 1. (C-F) Single-cell analysis revealed the distinct cell types of the tissue-specific serine proteases, including PRSS27, PRSS57, TMPRSS6 and TMPRSS15. All single-cell plots here were screenshots from the HPA database

According to the annotation of the human genome, a total of 24 trypsin-like serine proteases and 18 TTSPs were included into our integrated analysis. The expression data (RPKM values) for the serine protease genes were obtained by quantitative transcriptomic sequencing (RNA-Seq) data from the Human Protein Atlas (HPA) database [20]. Herein, we used the dot plots to reflect the landscape of expression pattern of 42 serine protease genes across human tissues (Fig. 1a and b). Based on the differences in expression patterns, serine proteases can be further divided into two categories, including non-tissue-specific serine proteases and tissue-specific serine proteases. Several PRSSs (such as PRSS8/prostasin, PRSS12/motopsin, PRSS22/tryptase ε, PRSS23, PRSS16, PRSS33, and PRSS36) and 5 TTSPs (HPN, TMPRSS2/3/4, TMPRSS9, and ST14) were none-tissue-specific serine proteases that are widely expressed in human tissues.

The expression pattern of tissue-specific serine proteases was further investigated by single-cell analysis. The cell types expressing these tissue-specific serine proteases were further identified using the available single-cell data in the HPA dataset. PRSS27, also known as marapsins or pancreasin, was specifically expressed in the esophagus squamous epithelial cells (Fig. 1c). Bone marrow-specific serine protease PRSS57 was specifically expressed in erythroid cells (Fig. 1d). Liver-specific serine protease TMPRSS6, also known as matriptase-2, was specifically expressed in the hepatocytes (Fig. 1e). TMPRSS15, also known as enteropeptidase, was specifically expressed in duodenal proximal enterocytes (Fig. 1f).

### The testis-specific serine protease PRSS56 is frequently upregulated in cancers

**Fig. 2:**
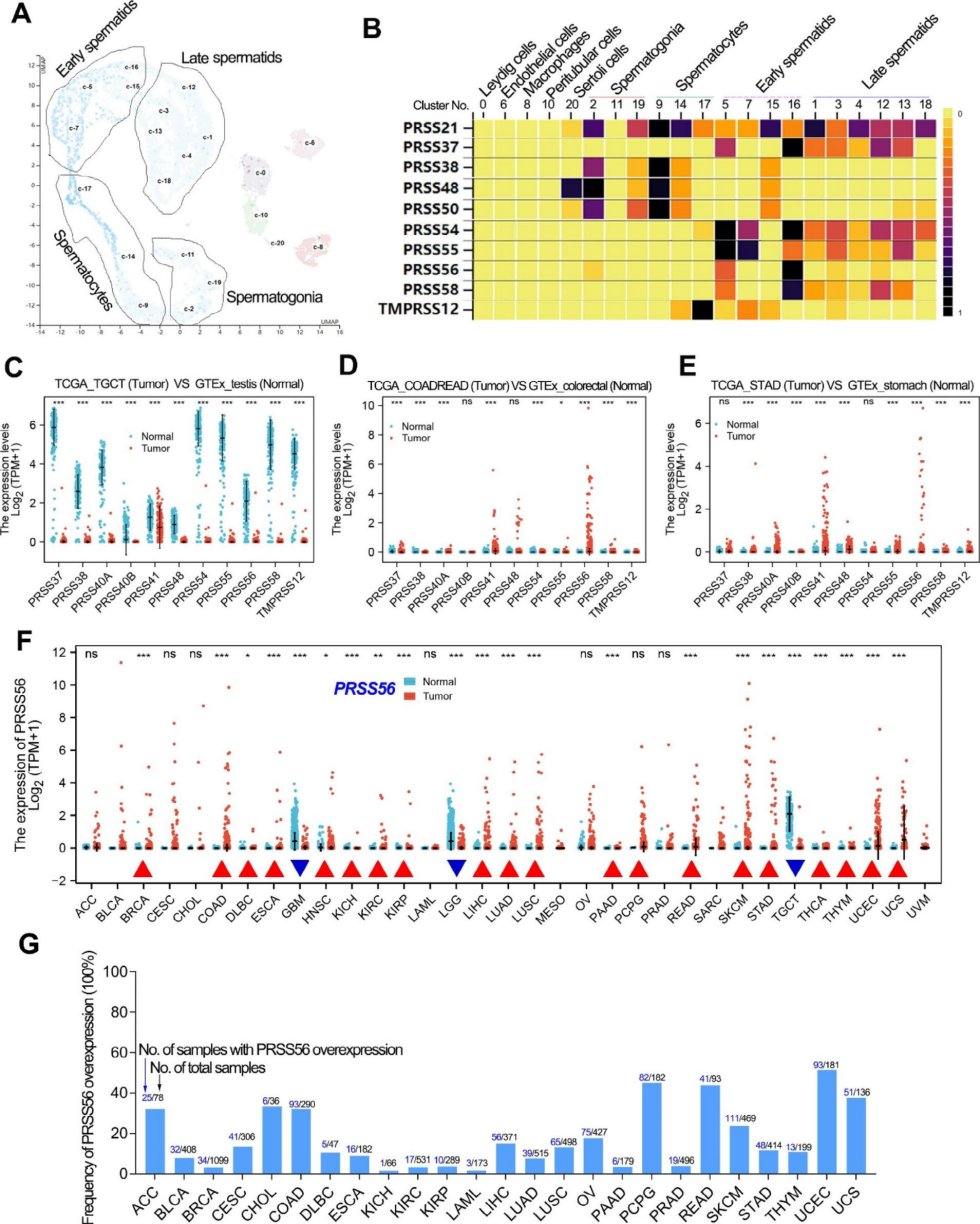
Serine protease PRSS56 was frequently overexpressed in cancers. (A) Single-cell analysis revealed that there were nine cell types in the normal testis tissue. The single-cell plot here was screenshots from the HPA database. (B) Serine proteases that can be detected expression data by single cell RNA sequencing were shown in the plot. The single-cell heat plots of different serine proteases were screenshots from the HPA database. (C**)** The testis-specific serine proteases were significantly downregulated in testis cancer. (D, E) Most of the testis-specific serine protease genes were significantly upregulated in GC and CRC. (F) The expression pattern of testis-specific serine protease PRSS56 was analyzed in pan-cancer. (G) The frequency of PRSS56 overexpression in pan-cancer was analyzed. The expression data (TPM value) of PRSS56 greater than 0.1 was considered overexpressed

According to the gene expression analysis in Fig. 1, there were at least 13 serine proteases that specifically expressed in testis, including the well-known serine protease testisin (also known as PRSS21). Single-cell analysis based on the HPA datasets contained nine cell types/clusters in the human testis tissue (Fig. 2a). Testisin/PRSS21 was widely expressed in the spermatogonia, spermatocytes, early spermatids and late spermatids (Fig. 2b). The testis-specific serine proteases, PRSS54, PRSS55, PRSS56 and PRSS58 were mainly expressed in early spermatids, while PRSS38, PRSS48 and PRSS50 were mainly expressed in spermatocytes and spermatogonia (Fig. 2b).

Interestingly, all the testis-specific serine proteases were significantly downregulated in testis cancer (Fig. 2c). On the contrary, the testis-specific serine proteases, such as PRSS56 and PRSS41, were greatly reactivated in stomach cancer and colorectal cancer (Fig. 2d, e). Notably, PRSS56 was the most significantly up-regulated testis-specific serine protease in gastric cancer. Thus, we further explored the PRSS56 expression pattern in pan-cancer. The pan-cancer analysis showed that PRSS56 expression was significantly upregulated in various cancers, especially GC and CRC (Fig. 2f). In addition, we also analyzed the frequency of PRSS56 overexpression in pan-cancer (Fig. 2g). Particularly, the frequency of PRSS56 overexpression in gastrointestinal cancer was 8.8% in esophagus cancer, 11.6% in gastric cancer, 32.1% in colon cancer, 44.1% in rectal cancer, and 15.1% in liver cancer, respectively.

### PRSS56 is a novel CT antigen that activated by DNA methyltransferase inhibitors

**Fig. 3:**
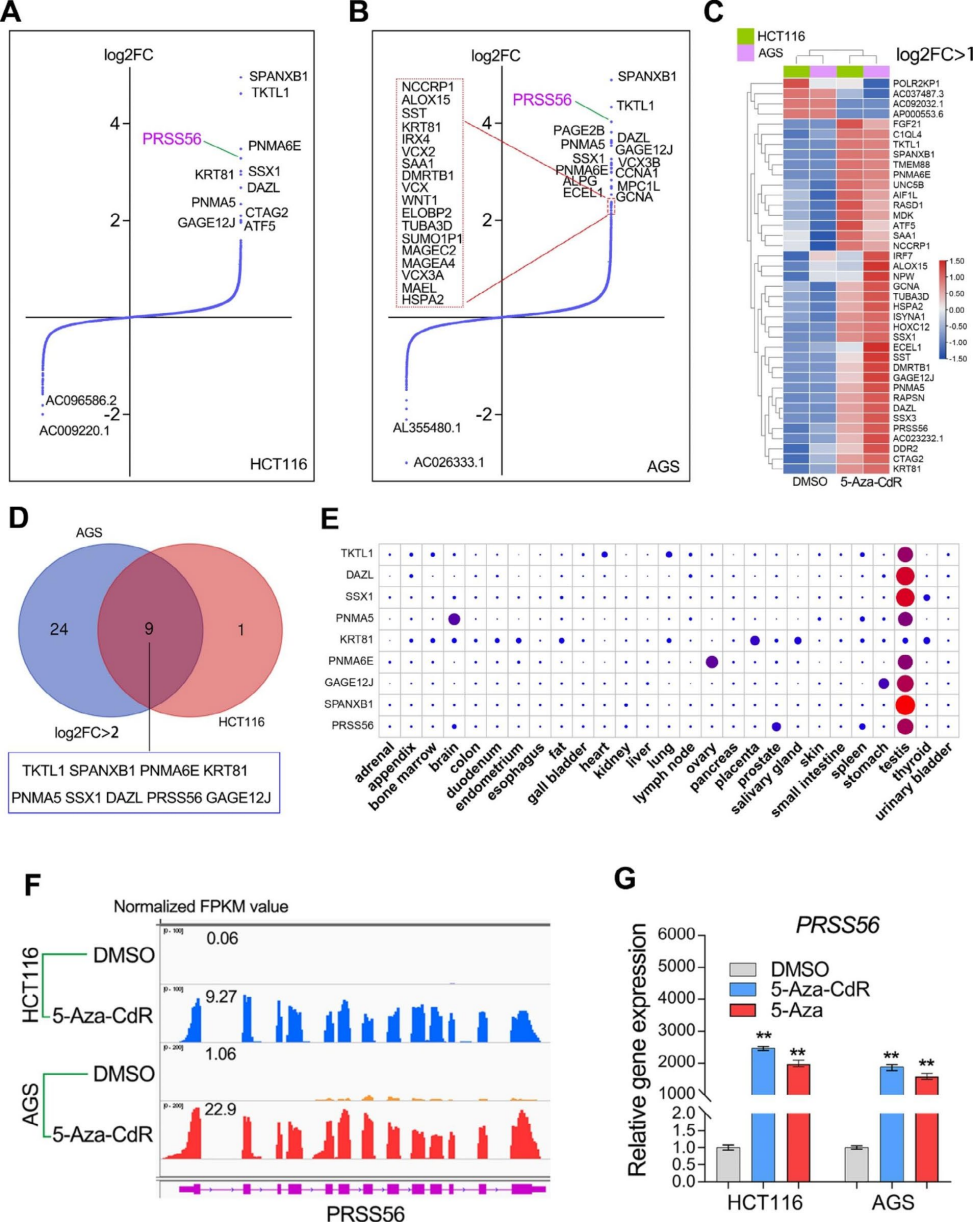
Serine protease PRSS56 is a novel CT antigen that reactivated by DNA methyltransferase inhibitor. (A and B) The RNA sequencing studies (GSE215214) were conducted in AGS and HCT116 cell lines exposure to the DNA methyltransferase inhibitor 5-Aza-CdR (5 µM). The Gene expression fold changes (log2FC value) were shown in the volcano plots. (C) The genes with the most significant expression fold changes (log2FC > 1) after 5-Aza-CdR treatment were displayed in the heat map. (D) There were nine genes with the most significant upregulation (log2FC > 2) after 5-Aza-CdR treatment were shown in the plot. (E) PRSS56 was a novel CT antigen that activated by 5-Aza-CdR. (F, G) RNA-seq analysis and qRT-PCR assay together confirmed that PRSS56 was reactivated by 5-Aza-CdR in both AGS and HCT116 cell lines

Increasing studies have uncovered the essential roles of DNA hypomethylation in activating the expression of CT genes [21]. Thus, in order to further confirm whether PRSS56 is a CT gene or not, RNA sequencing studies were performed in human cancer cell lines after exposure to the well-known DNA methyltransferase inhibitor (5-AZA-CdR). Given PRSS56 was greatly overexpressed in GC and CRC, we herein selected a gastric cancer cell line AGS and a colorectal cancer cell line HCT116 for DNA methyltransferase inhibitor treatment (Fig. 3a, b).

According to the RNA-seq analysis, 5-Aza-CdR treatment in HCT116 and AGS cell lines resulted in the great upregulation (log2 FC > 1) of 35 genes (Fig. 3c). Among them, 9 genes have the most significant changes in expression after 5-AZA-CdR exposure (log2 FC > 2), including the testis-specific SPANXB1, DAZL, TKTL1, SSX1, PNMA5, PNMA6E and GAGE12J and PRSS56 (Fig. 3d, e). SPANXB1, DAZL, TKTL1, SSX1, PNMA5, PNMA6E and GAGE12J are well-known CT antigens, while PRSS56 is a novel CT antigen that has not been reported. RNA-seq analysis showed that PRSS56 was greatly reactivated by 5-Aza-CdR in both HCT116 and AGS cell lines (Fig. 3e). Quantitation RT-PCR assay further confirmed that PRSS56 expression was significantly up-regulated after treatment with 5-Aza or 5-Aza-CdR in the HCT116 and AGS cell lines (Fig. 3f, g).

### PRSS56 expression was negatively correlated with promoter DNA methylation but positively correlated with gene body methylation

**Fig. 4:**
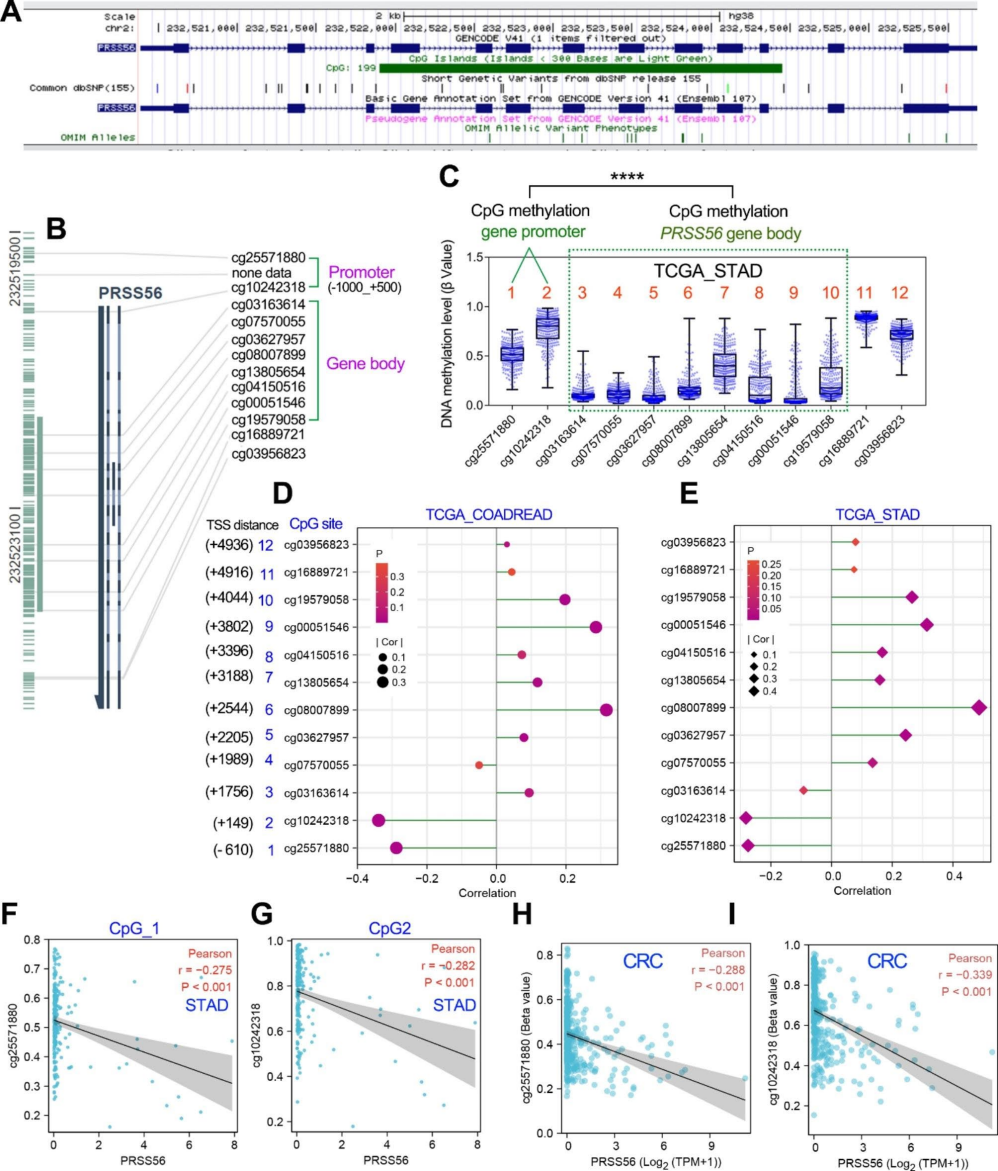
The correlation analysis between PRSS56 expression and DNA methylation level in GC and CRC. (A) The UCSC web tool showed that there is a CpG island in the PRSS56 gene body DNA. (B) The CpG sites in PRSS56 promoter and gene body DNA were displayed using the MEEXPRESS web tool. (C) The methylation level of PRSS56 promoter DNA is significantly higher than that of gene body DNA. The methylation data of each CpG site was obtained from TCGA_STAD samples. (D, E) PRSS56 expression was positively correlated with promoter DNA methylation and negatively correlated with gene body DNA methylation in GC and CRC. The methylation data of each CpG site and PRSS56 expression data were obtained from TCGA database. (F-I) PRSS56 expression was negatively correlated with the methylation level of the CpG sites in GC and CRC

Since PRSS56 expression was significantly up-regulated by DNA methyltransferase inhibitors, we speculated that DNA methyltransferase inhibitors may activate PRSS56 expression by reducing the methylation level of the promoter DNA. According to UCSC web site, there is a CpG island in the PRSS56 gene body (Fig. 4a). The MEXPRESS web tool further confirmed that there were 2 CpG sites in PRSS56 promoter DNA and 8 CpG sites in the gene body region (Fig. 4b). Interestingly, DNA methylation analysis showed that the methylation level in PRSS56 promoter was higher than the methylation level in PRSS56 gene body (Fig. 4c).

Next, the correlation analysis between PRSS56 expression (TPM value) and the DNA methylation levels (β value) of different CpG sites relevant to PRSS56 was performed in GC and CRC using the TCGA database. Notably, PRSS56 expression was positively associated with their gene body DNA methylation levels in GC and CRC (Fig. 4d, e), but was negatively associated with their promoter DNA methylation levels in GC and CRC (Fig. 4f-i), suggesting that PRSS56 expression was negatively regulated by their promoter DNA methylation.

### PRSS56 expression was negatively regulated by promoter DNA methylation

**Fig. 5:**
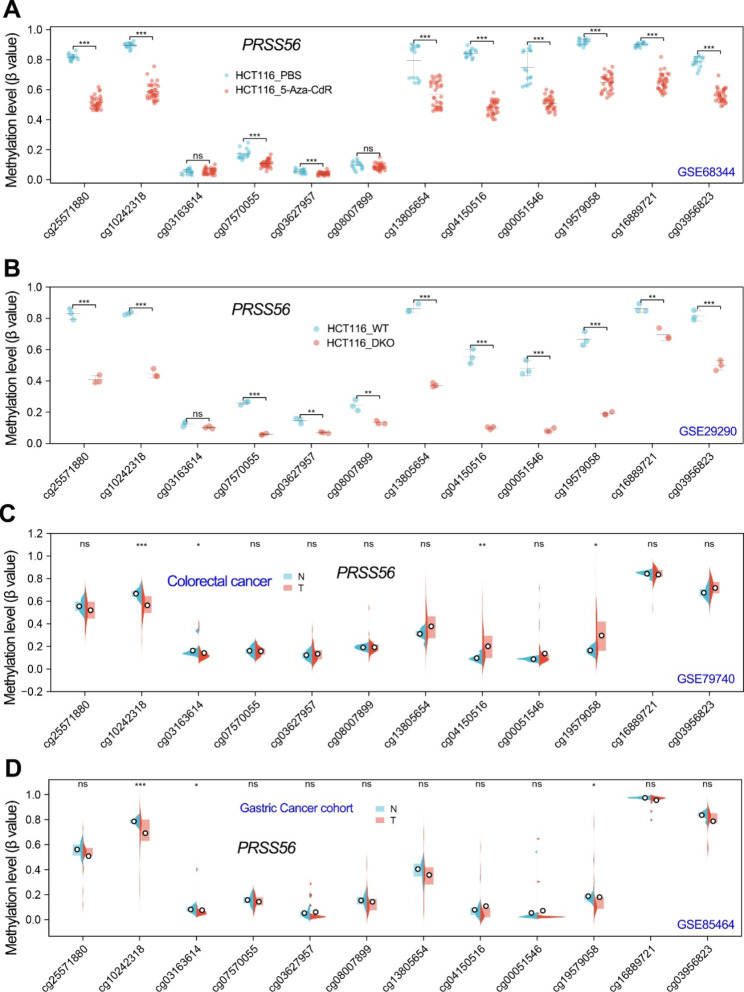
The DNA methylation level of PRSS56 gene in GC and CRC. (A) The DNA methylation level (β value) of PRSS56 in HCT116 cells treated with 5-Aza-CdR was analyzed using GSE68344 dataset. (B) The DNA methylation level (β value) of PRSS56 in wildtype (WT) and DNMT1 and DNMT3B double KO (DKO) HCT116 cells treated with 5-Aza-CdR was analyzed using GSE29290 dataset. (C, D) The DNA methylation level (β value) of PRSS56 in normal and tumor tissues in GC and CRC patients using GSE79740 and GSE85464 dataset

Based on the current findings, we speculated that DNA methyltransferase inhibitors may up-regulate PRSS56 expression by reducing promoter DNA methylation level. Therefore, we further analyzed the effect of 5-Aza-CdR on the DNA methylation level of PRSS56 gene. Duymich and colleagues have deposited a DNA methylation dataset (GSE68344) in HCT116 cell line to GEO database [22]. The GSE68344 dataset contained DNA methylation data of 450 K CpG sites in colon cancer cell line HCT116 with/without 5-Aza-CdR treatment. After analysis of the DNA methylation level of each CpG sites in PRSS56 gene, we found that 5-Aza-CdR treatment significantly decreased the DNA methylation level of CpG sites in PRSS56 promoter and gene body (Fig. 5a), suggested that 5-Aza-CdR activated PRSS56 expression by reducing PRSS56 promoter DNA methylation. Additionally, the GSE29290 dataset contained DNA methylation data of 450 K CpG sites in wildtype HCT116 and DNMT1/DNMT3B double knockout (DKO) HCT116 cell line. Analysis of the PRSS56 gene methylation in GSE29290 showed that knockout of DNMT1/DNMT3B significantly decreased the PRSS56 promoter DNA methylation level in HCT116 cell line (Fig. 5b). This result implied that DNA methyltransferase DNMT1/DNMT3B may be required for PRSS56 promoter DNA methylation in GC and CRC.

Our above results have confirmed that PRSS56 was significantly up-regulated in GC and CRC. Thus, we speculated that the PRSS56 overexpression in GC and CRC may be due to its promoter DNA hypomethylation. To confirm this speculation, we further analyzed the DNA methylation level of PRSS56 in GC and CRC. The GSE85464 dataset contained the DNA methylation data of 450k CpG sites in 19 paired gastric cancer samples and GSE79740 dataset contained 44 colon cancer samples and 10 normal colon tissue samples. As expected, DNA methylation analysis in GSE85464 and GSE79740 showed that the DNA methylation level at the cg10242318 CpG site in PRSS56 promoter was significantly decreased in GC and CRC (Fig. 5c and d). These results firmly suggested that it is the reduced promoter DNA methylation that resulted in the activation of PRSS56 expression in GC and CRC.

### PRSS56 overexpression promoted tumor cell proliferation, migration and invasion in GC and colorectal cancer

**Fig. 6:**
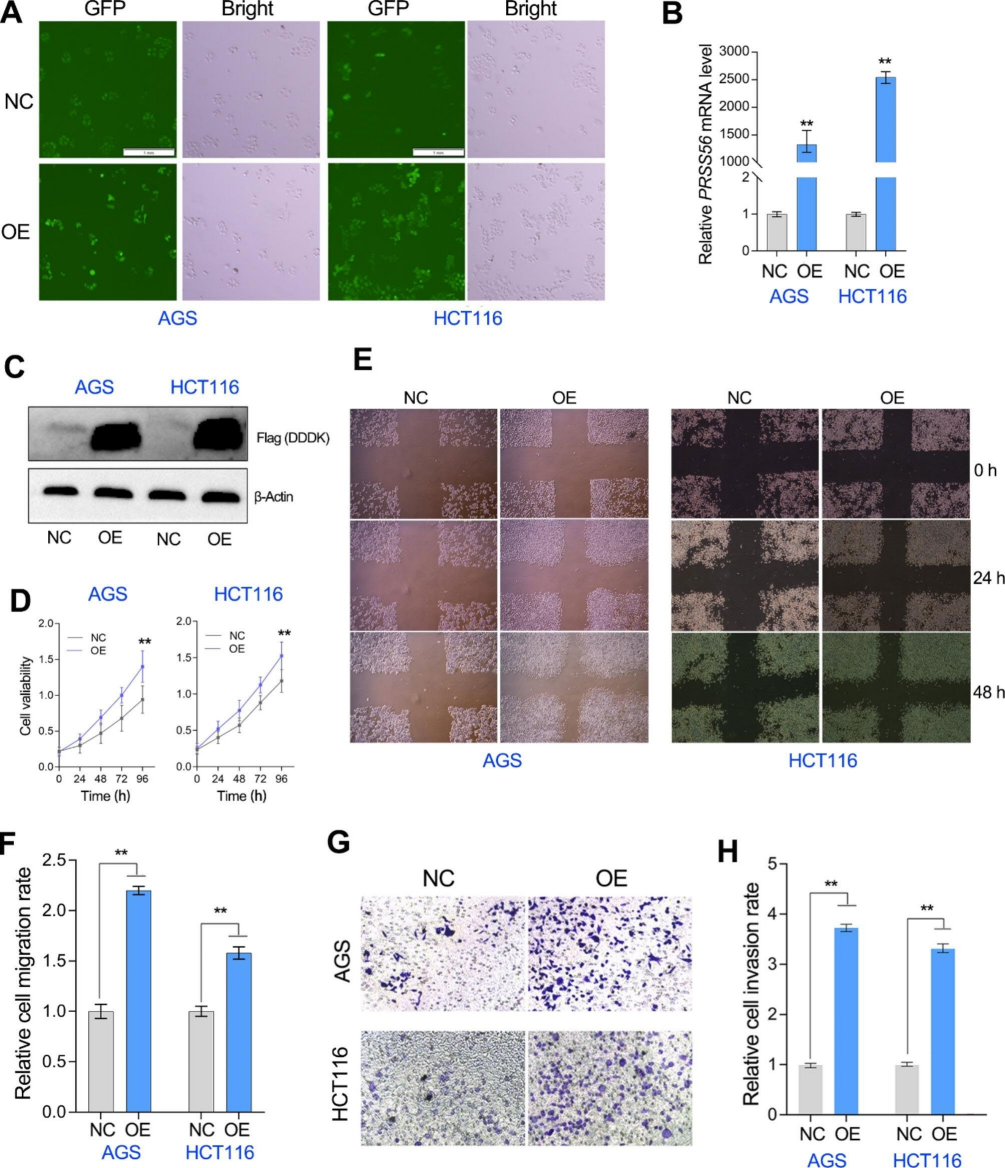
PRSS56 overexpression promoted tumor progression in GC and CRC. (A) The GC and CRC cell lines stably overexpressing PRSS56 were constructed by lentivirus method. (B, C) The overexpression efficiency of PRSS56 in the relevant cell lines was measured by the qRT-PCR and western blotting assays. (D) Overexpression of PRSS56 promoted tumor cell proliferation in GC and CRC. (E, F) Overexpression of PRSS56 promoted tumor cell migration at indicated hours (24 and 48 h) in GC and CRC. (G, H) Overexpression of PRSS56 promoted tumor cell invasion in GC and CRC. **P < 0.01

To understand the significance of PRSS56 overexpression in gastrointestinal cancer, gain-of-function studies regarding PRSS56 were performed in HCT116 and AGS cell lines using the lentiviral method (Fig. 6a). Our sequential qRT-PCR and western blotting assays further showed that PRSS56 was significantly overexpressed in HCT116 and AGS cell lines (Fig. 6b, c). Notably, according to qRT-PCR assay, PRSS56 gene was lowly expressed in the HCT116 and AGS cell lines. Cell proliferation assay showed that PRSS56 overexpression significantly accelerated the growth of HCT116 and AGS cell lines, suggested PRSS56 positively regulated gastrointestinal cancer cell proliferation (Fig. 6d). Wounding healing assay also confirmed that PRSS56 overexpression significantly promoted the migration of HCT116 and AGS cell lines (Fig. 6e, f). Similarly, the transwell assay showed that PRSS56 overexpression obviously enhanced the invasion of HCT116 and AGS cell lines (Fig. 6g, h). These results suggested that PRSS56 overexpression functioned as an oncogene in gastrointestinal cancer progression.

### PRSS56 overexpression exhibits high immunogenicity and promoted gastric and colorectal cancer via PI3K/AKT axis

**Fig. 7:**
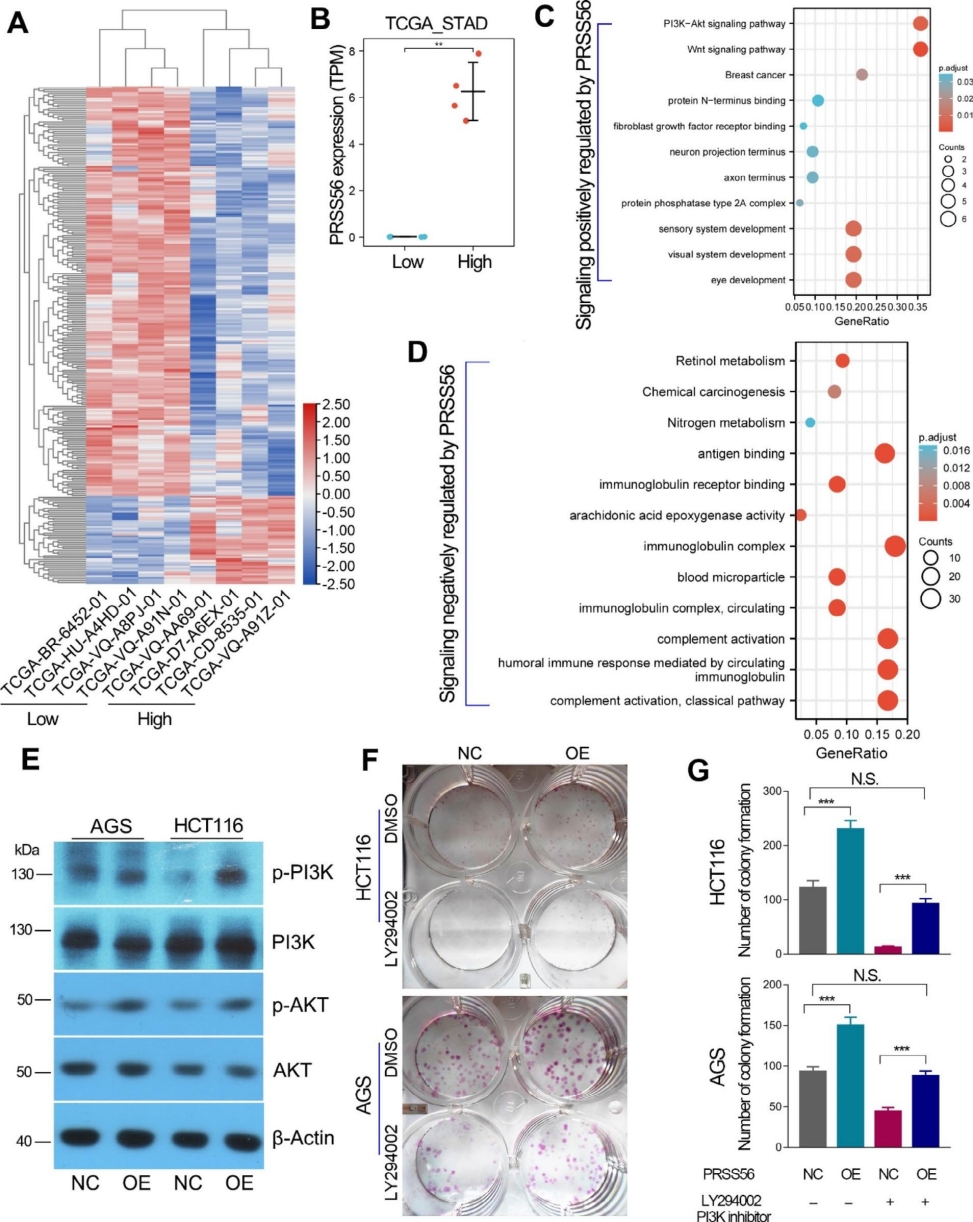
PRSS56 promotes GC and CRC via activation of PI3K/AKT axis. (A, B) The differences in transcriptome between GC patients with PRSS56 overexpression and GC patients without PRSS56 expression. (C) The genes upregulated in GC patients with PRSS56 overexpression were used to conducted KEGG pathway analysis. (D) The genes downregulated in GC patients with PRSS56 overexpression were used for conducting KEGG pathway analysis. (E) Western blotting assay confirmed that PRSS56 overexpression activated the PI3K/AKT axis. (F, G) Rescue colony formation assay using PI3K inhibitor (LY294002, 10 µM) confirmed that PRSS56 overexpression promoted GC and CRC cells proliferation through PI3K/AKT axis. **, P < 0.01

In order to explore the potential molecular mechanism of PRSS56 driving cancer progression, we compared the transcriptome differences between four patients with high PRSS56 expression and four patients with low PRSS56 expression in the TCGA_STAD cohort (Fig. 7a, b). Then, the genes positively or negatively correlated with PRSS56 expression were used to conduct GO/KEGG pathway analysis, respectively. Previous studies have confirmed a critical role of PRSS56 in eye development [23]. Consistently, our results also showed that PRSS56 overexpression was involved in eye development, visual system development and sensory system development, and activated the PI3K/AKT and Wnt/beta-catenin signaling pathways (Fig. 7c). As a CT antigen, PRSS56 has high immunogenicity in cancers. The KEGG pathway analysis also showed that PRSS56 was negatively associated with complement activation, antigen and immunoglobulin receptor binding (Fig. 7d).

As PI3K-AKT signaling pathway is activated in the GC tissues with high PRSS56 expression, we considered that PRSS56 might promote GC and CRC progression via activating PI3K/AKT axis. To further verify this speculation, western blotting assays were performed to examine the effect of PRSS56 overexpression on PI3K/AKT axis in GC and CRC cell lines. As expected, PRSS56 overexpression significantly activated PI3K/AKT signaling pathway in GC and CRC (Fig. 7e). More importantly, rescue colony formation assays further identified that the promoting effect of PRSS56 overexpression on GC and CRC cell proliferation can be partially can be partially or even completely eliminated after incubation with the PI3K inhibitor LY294002 (Fig. 7f, g).

**Fig. 8:**
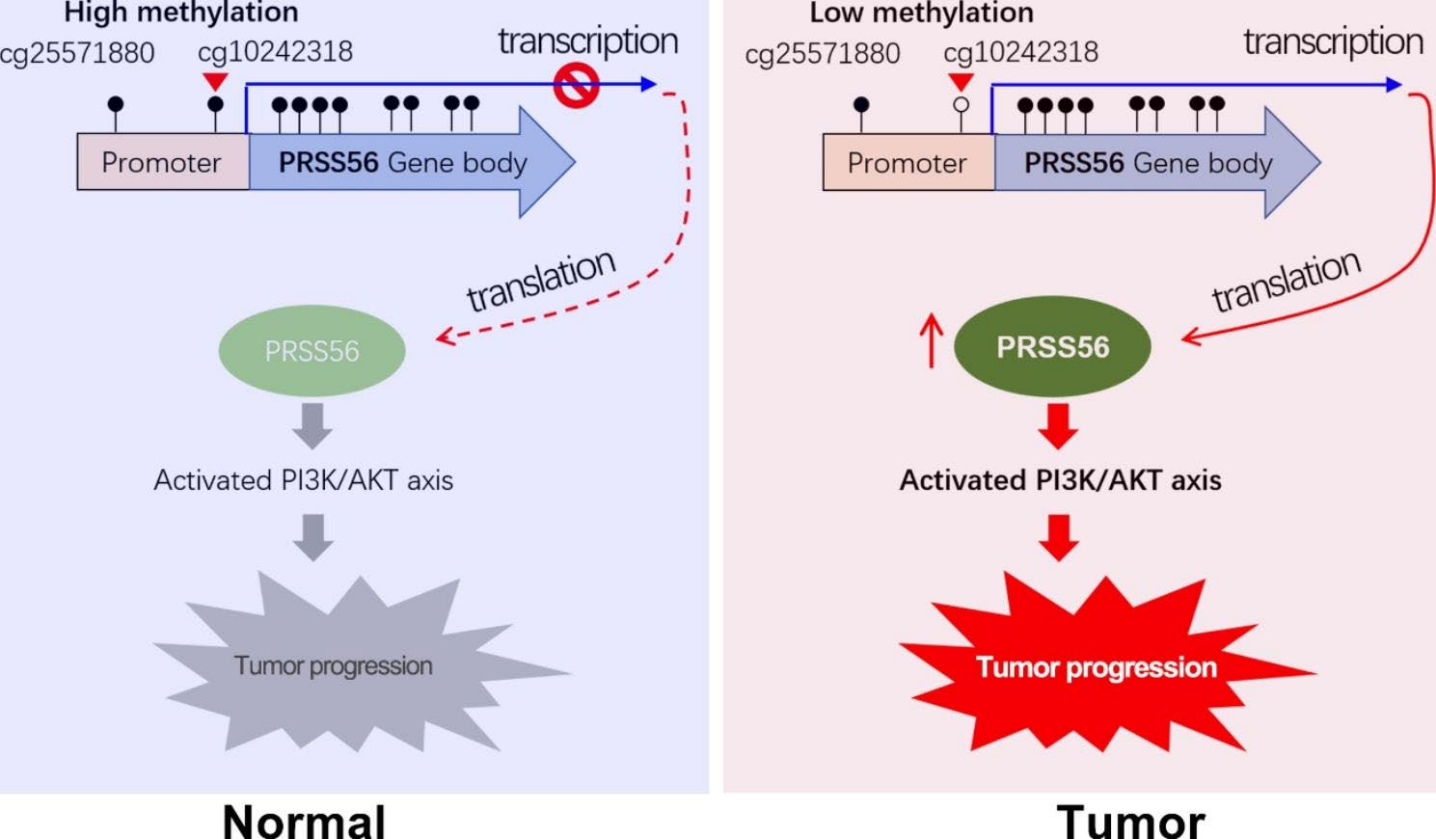
The working model of the CT antigen PRSS56 in GC and CRC. Briefly, PRSS56 was a novel CT antigen specifically expressed in normal testis, and its expression was frequently reactivated in diverse tumors, especially gastrointestinal cancer. In GC and CRC, PRSS56 overexpression was due to its reduced methylation level of PRSS56 promoter at CpG site cg10242318. In turn, PRSS56 overexpression promoted GC and CRC progression by activating PI3K/AKT axis

In summary, PRSS56 was identified as a novel cancer-testis antigen, which is overexpressed in numerous cancers, especially gastrointestinal cancers. Importantly, our finding suggested the overexpression of PRSS56 in cancers is probably due to its promoter DNA hypomethylation. Particularly, in vitro experiments indicated that PRSS56 exerts oncogenic roles in GC and CRC by activating PI3K/AKT signaling (Fig. 8).

## Discussion

Cancer/testis (CT) antigens/genes are immunogenic in cancer patients, exhibit highly tissue-restricted expression, and are considered promising target molecules for cancer vaccines [3]. In normal human tissues, the vast majority of CT genes are selectively expressed in testis, but almost none in other tissues [24]. However, the expression pattern of CT genes is not applicable to tumor tissues, because their expression is reactivated in tumors other than testis cancer [6, 25]. Because of this, CT antigens present potential for use as biomarkers and targets for immunotherapy [26–28]. To date, a large number of CT gene families have been identified and their expression studied in numerous cancer types, such as the SSX gene family [29], the SPANXB gene family [30], the PNMA gene family [31], the GAGE gene family [32].

In this study, the testis-specific serine protease PRSS56 was identified as a novel CT antigen. The CT antigen PRSS56 was selectively expressed in early spermatids of normal testis and widely upregulated in various cancers other than testis and brain cancer, especially in gastrointestinal cancer. Accumulating evidence has shown that DNA hypomethylation is an essential factor leading to the upregulation of CT genes in cancers [33]. Our RNA-Seq analysis also found that 5’-Aza-CdR exposure resulted in overexpression of multiple CT antigens in gastric cancer and colorectal cancer, including SSX1, TKTL1, SPANXB1, PNMA5, PNMA6E, GAGE12J, and PRSS56. Moreover, it has been found that in GC and CRC, the expression of PRSS56 was positively correlated with the methylation level of gene body CpG sites and negatively associated with the methylation level of the CpG sites in promoter DNA. Particularly, the methylation levels of the CpG site (cg10242318) in the promoter DNA play critical roles in regulating PRSS56 expression. Our finding highlights that the methylation level of the CpG site (cg10242318) in the promoter DNA of PRSS56 was significantly reduced in GC and CRC, which resulted in the overexpression of PRSS56 in GC and CRC. By extension, the overexpression of PRSS56 in other cancers may also be caused by the hypomethylation of its promoter DNA.

Previous studies have revealed that PRSS56 plays a critical role in neurogenesis and eye development [34, 35]. Mutations in the PRSS56 gene are a cause of ocular angle defects and autosomal recessive posterior microphthalmos [23, 36]. However, the biological function of PRSS56 in cancers remains unknown. In this study, we first confirmed the oncogenic roles of PRSS56 in gastric and colorectal cancer. PRSS56 overexpression significantly promoted GC and CRC cells proliferation, migration and invasion. In addition, the GO/KEGG analysis has confirmed that PRSS56 overexpression exhibits high immunogenicity and was positively associated with PI3K/AKT axis in gastric cancer patients. In consistent, overexpression of PRSS56 activated PI3K/AKT signaling axis in GC and CRC, suggesting that PRSS56 promotes GC and CRC via PI3K/AKT axis.

## Conclusions

In summary, serine protease PRSS56 is identified as a novel CT gene or antigen. The expression of PRSS56 is negatively regulated by its promoter DNA methylation. The CT antigen PRSS56 is frequently overexpressed in cancers, especially in gastrointestinal cancer. Mechanismly, the overexpression of PRSS56 in GC and CRC is due to the decreased methylation level of CpG site (cg10242318) in its promoter DNA. More importantly, our finding highlights that overexpression of PRSS56 promoted GC and CRC progression through activation of PI3K/AKT axis (Fig. 8).

## Materials and methods

### Gene expression analysis

The gene expression data in different human normal tissues was obtained from the National Center for Biotechnology Information (NCBI) website, as we mentioned previously [37–39]. The gene expression data and corresponding clinical information and DNA methylation data in cancers were obtained from The Cancer Genome Atlas (TCGA) dataset. The expression level of per gene was measured by the transcripts per million (TPM) value. The pan-cancer analysis of PRSS56 was performed using XIANTAO web tool. The single-cell analysis of serine proteases was obtained from the Human Protein Atlas (HPA) project [40].

### DNA methylation analysis

The information of CpG sites in promoter or gene body of PRSS56 were obtained from the MEXPRESS dataset and UCSC website. The DNA methylation level of each CpG site was measured by the normalized beta value using Illumina HumanMethylation450 BeadChip (Platform GPL13534). For GC cohort, the DNA methylation level of each CpG site was obtained from GSE85464 dataset. For CRC cohort, the DNA methylation level of each CpG site was obtained from GSE79740 dataset. The DNA methylation level of each CpG site after 5-Aza-CdR treatment or knockout of DNMTs was obtained from GSE29290 and GSE68344 dataset. The correlation of PRSS56 expression and methylation level of each CpG site (β value) was analyzed using Pearson Method.

### Cell culture and cell transfection

The human cancer cell lines (AGS and HCT116) were purchased from the Shanghai Cell Bank of Chinese Academy of Sciences. All the cell lines were cultured in DMEM medium containing 10% fetal bovine serum, 100 U/mL penicillin,100 U/mL streptomycin and 0.03% glutamine at 37 ^o^C in 5% CO2. For stable overexpression of PRSS56, the lentiviruses of oe_PRSS56 were purchased from Genepharma. Lentiviral transfection was performed according to the manufacturer’s instructions. At the indicated time points, the cells were harvested for mRNA and protein analysis as well as for other assays. Wound healing assay and transwell invasion assay were performed as we previously described [41].

### DNA methyltransferase inhibitor treatment

Briefly, cell lines were seeded into 6-well plates and grown as usual. When the cell plating density reached 70–80%, cells were treated with 5-aza-2’-deoxycytidine (5-AZA-CdR, 5 µM) or 5-Azacytidine (5-Aza, 5 µM) for 48 h. At the indicated time points, the cells were harvested for mRNA analysis as well as RNA-seq studies. The 5-aza-CdR (Catalog No., A1906) and 5-Aza (Catalog No., A1907) were purchased from APEXBIO company (Houston, USA) and dissolved in Dimethyl sulfoxide (DMSO), respectively at a final concentration of 100 nM to prepare a suitable stock solution. DMSO was purchased from Merck Co. (Darmstadt, Germany).

### RNA isolation and quantitative RT-PCR

The qRT-PCR assay was performed as previously described [42–44]. Briefly, Total RNA was extracted using Trizol reagent (Invitrogen, USA). Reverse transcription was performed to obtain cDNA by using the PrimeScript^™^ RT reagent Kit (Perfect Real Time, Takara). The qPCR protocol was using One Step TB Green PrimeScript^™^ RT-PCR Kit II (Takara) according to the manufacturer’s instructions. The qPCR analysis was conducted on Bio-Rad CFX Manager 3.1 real-time PCR system. The primers used in this study were listed as below: PRSS56-F, TTCATGAGGTCCTGGCAGAT, PRSS56-R: GGTACCTGAGGGTTGAGTGG; ACTIN-F, ATCGTCCACCGCAAATGCTTCTA, ACTIN-R, AGCCATGCCAATCTCATCTTGTT.

### Western blotting assay

The western blotting assay was performed as previously described [45]. The antibodies of AKT (60203-2-Ig), p-AKT (28731-1-AP), p-PI3K (AP0854) and PI3K (20584-1-AP) were purchased from Abclonal company (Wuhan, China) and Proteintech company (Wuhan, China). Briefly, cells were lysed in RIPA buffer added 1 mM PMSF. Approximately 50–100 µg of total protein was electrophoresed through 10% SDS polyacrylamide gels and were then transferred to a PVDF membrane. After blocking with 5% skimmed milk at 4 °C for 1 h, the membrane was incubated with primary antibody at 4 °C overnights. The blots were then washed and incubated with horseradish peroxidase (HRP)-conjugated secondary antibody (1: 10,000, Earthox) for 1.5 h at room temperature. Detection was performed by using a SuperLumia ECL HRP Substrate Kit (Abbkine) and visualized using a Bio-Rad Imaging System (USA).

### RNA sequencing

RNA sequencing studies were performed as previously described [45–47]. After 48 h treatment with 5-Aza-CdR, total RNA of HCT116 and AGS cells were extracted to perform RNA sequencing. A total amount of 1.5 µg RNA per sample was used as input material for the RNA sample preparations. The whole step of library construction and sequencing was performed at Shanghai Lifegenes Technology Co., Ltd. The RNA-seq data was uploaded on the GEO section of NCBI web server. The GEO accession number was GSE215214.

### Rescue assay

PI3K inhibitor LY294002 was purchased from Biovision (San Francisco, CA, USA). The concentration of LY294002 used in this study was 10 µM. Briefly, the effect of PI3K inhibitor and PRSS56 overexpression on cell colony formation was tested by clonogenic assay. Briefly, about 2000 cells (oe_NC and oe_PRSS56)were seeded in a 12-well. The medium containing 0.5% of DMSO served as a negative control. The medium containing 10 µM LY294002 served as PI3K treatment. After incubation for eight days, the colonies were then rinsed twice and fixed with 4% paraformaldehyde solution. Colonies were stained with a 0.1% aqueous crystal violet solution for 15 min and washed twice with sterile deionized water. Then, the number and area of clones were counted. Each group undergoes 4–6 biological replicates.

### Statistical analysis

For gene expression or DNA methylation analysis of different subtypes of GIC, the P values were estimated using Mann–Whitney nonparametric test. Pearson correlation analysis was used for the correlation test of the two groups of data. The other experiments were used unpaired t-test or one-way ANOVA test. For quantitative RT-PCR, a minimum of triplicates per group and repetition of at least three times was applied to achieve reproducibility. All tests with p values less than 0.05 were considered to be statistically significant.

## Data Availability

The datasets supporting the conclusions of this article are included within the article and its additional files.

## Abbreviations

GC: Gastric cancer
CRC: Colorectal cancer
TCGA: The Cancer Genome Atlas
TPM: Transcripts per million
qRT-PCR: Quantitative reverse transcription Polymerase Chain Reaction
GEO: Gene Expression Omnibus
TTSP: Type II transmembrane serine proteases
PRSS: Trypsin-like serine proteases
CT: Cancer-testis
5-AZA-CdR: 5-aza-2’-deoxycytidine
5-Aza: 5-Azacytidine
HPA: The Human Protein Atlas
ACC: Adrenocortical carcinoma
BLCA: Bladder Urothelial Carcinoma
BRCA: Breast invasive carcinoma
CESC: Cervical squamous cell carcinoma and endocervical adenocarcinoma
CHOL: Cholangio carcinoma
COAD: Colon adenocarcinoma
DLBC: Lymphoid Neoplasm Diffuse Large B-cell Lymphoma
ESCA: Esophageal carcinoma
GBM: Glioblastoma multiforme
HNSC: Head and Neck squamous cell carcinoma
KICH: Kidney Chromophobe
KIRC: Kidney renal clear cell carcinoma
KIRP: Kidney renal papillary cell carcinoma
LAML: Acute Myeloid Leukemia
LGG: Brain Lower Grade Glioma
LIHC: Liver hepatocellular carcinoma
LUAD: Lung adenocarcinoma
LUSC: Lung squamous cell carcinoma
MESO: Mesothelioma
OV: Ovarian serous cystadenocarcinoma
PAAD: Pancreatic adenocarcinoma
PRAD: Prostate adenocarcinoma
READ: Rectum adenocarcinoma
SARC: Sarcoma
SKCM: Skin Cutaneous Melanoma
STAD: Stomach adenocarcinoma
TGCT: Testicular Germ Cell Tumors
THCA: Thyroid carcinoma
THYM: Thymoma
UCEC: Uterine Corpus Endometrial Carcinoma
UCS: Uterine Carcinosarcoma
UVM: Uveal Melanoma
